# Knowledge and attitudes among preschools staff in Shanghai, China, regarding epilepsy

**DOI:** 10.1186/s12887-020-02376-3

**Published:** 2020-10-13

**Authors:** Liyan Qiu, Lixiao Shen, Junli Wang, Fang Ren, Mingyu Xu, Fan Jiang, Xiaoyang Sheng, Fei Li, Feng Li

**Affiliations:** 1 Department of developmental behavioral pediatric & children healthcare, MOE-Shanghai Key Laboratory of Children’s Environmental Health, Xinhua Hospital, School of Medicine, Shanghai Jiao Tong University, 1665 Kongjiang Rd, Shanghai, 200092 China; 2 Department of Developmental and Behavioral Pediatrics, Shanghai Pediatric Translational Research Institute, Shanghai Children’s Medical Center, Shanghai Jiao Tong University, MOE-Shanghai Key Laboratory of Children’s Environmental Health, 1678 Dongfang Rd, Shanghai, 200127 China

**Keywords:** Epilepsy, Preschool staff, First-aid knowledge, Attitudes, Training

## Abstract

**Background:**

Epilepsy is one of the most common neurological problems among children. The aim of this survey was to assess the knowledge and attitude among preschool staff in Shanghai regarding epilepsy.

**Methods:**

A cross-sectional survey was carried out among the staff at selected preschools. A stratified random sampling method was first used to identify suitable subjects. Data were obtained using a self-completed questionnaire. A standardized collection of demographic information was performed, and participants were given a questionnaire about their knowledge and attitudes regarding epilepsy.

**Results:**

A total of 1069 subjects completed the questionnaire. In this survey, 387 (36.2%) staff members had previously participated in related training. 17.6% of teachers knew how to provide appropriate first aid for seizures. Correct responses regarding first aid for seizures, such as laying the person on his or her side (24.9%), moving harmful objects out of the way (20.7%), protecting the head (36.1%), waiting until the seizure ends (7.9%), and dialing the emergency number (40.1%), were low. The staff members had different attitudes towards children with epilepsy: some subjects had a positive attitude, some had a negative attitude.

**Conclusions:**

The level of first-aid knowledge among preschool staff in Shanghai relevant to epilepsy was low. There is an urgent need to educate staff about epilepsy and appropriate first-aid practices for seizures.

## Background

Epilepsy is one of the most common neurological problems among children, with a worldwide prevalence of 5 ~ 10 people per 1000 people [1]. In Europe, the prevalence of epilepsy is 3.3 ~ 7.8 people per 100 people [2]. In China, epidemiological research has shown that the prevalence of epilepsy has been 5.95–8.75 people per 1000 people since 2000 [3]. There are more than 2 million people with epilepsy in China [4]. Seizures can occur suddenly and at any time, potentially causing severe harm or even death [5]. Children in preschool are one groups affected by epilepsy, and a negative attitude towards afflicted children can lead to physical injuries, social isolation and learning difficulties [6]. As most children spend the day in schools and situations requiring first aid are often encountered there, especially in preschool, these are important locations in which to focus on the management of epilepsy in children. The response time in emergency situations is critical, but the first aid provided must be performed properly to prevent further complications and to help save lives [7]. The likelihood of a successful cessation of a seizure is 96% if rescue medication is administered during the first 15 min of the seizure. Success rates decline to 57% if rescue medication is administered with longer delay [8, 9]. Therefore, administering appropriate and timely first aid to children after a seizure is vital and can potentially save lives.

In preschools, the person closest to the child and therefore the first to apply first aid is often a preschool staff member. Staff members in schools are often first-aid providers, and it is important to determine the current perceptions held by school staff concerning children’s epilepsy [10]. However, Okumura A et al. have indicated that school teachers have insufficient knowledge about epilepsy and hold negative attitudes, and such problems are too common in Japan [11] It is vital that preschool teachers are provided with necessary knowledge about safety precautions and first aid when dealing with epilepsy [9]. Therefore, it is important to investigate preschool teachers’ knowledge of epilepsy and attitudes towards children with epilepsy. However, there are few studies on the epilepsy knowledge of teachers in China, and the goal of the current survey was therefore to use questionnaires to evaluate preschool employees’ knowledge of epilepsy in children and attitudes towards children with epilepsy in order to promote the preschool teachers’ knowledge and training regarding epilepsy.

## Methods

### Subjects

This survey was designed as an across-sectional study from February 2020 to June 2020.

The research method is similar to our previous research, as previously described [12–14]. The survey involved stuff working in preschools in Shanghai, China. And preschools in China serve as both educational and childcare institutions. Preschools staff include teachers, healthcare providers (persons who have some medical knowledge but are generally not doctors), directors. There are 16 districts in shanghai consisted of eight urban areas and eight suburbs, including 1715 preschools. Eight districts were randomly selected including four urban areas and four suburbs. The number of staff members in each district selected to participate in the survey was based on the total number of staff members in the district. A stratified random sampling method was used to obtain 1125 subjects. And a cross-sectional survey was conducted using a self-filled questionnaire to assess the participant’s knowledge of epilepsy. The participants were not allowed to check any reference materials. All participants provided written informed consent. The Institutional Review Board of Shanghai Xinhua Hospital approved the survey, and the survey was conducted in accordance with the Helsinki Declaration.

### Procedures

The data were collected by a questionnaire, which was divided into two sections. Section A focused on the demographic information of the participants. Section B comprised 16 simple-choice questions on knowledge about the treatment of epilepsy that came from previously published papers [15–19]. Section B included the following aspects: (1) knowledge about the cognitive and behavioral characteristics of people with epilepsy (e.g., “Most children with epilepsy can go to public schools”); (2) knowledge about general knowledge for epilepsy (e.g., “Do you think epilepsy is a contagious disease?”); and (3) knowledge about the management of a seizure (e.g., “When you see a person having a seizure, can you provide emergency assistance?”) [19]. We ensured that participants answered all questions.

### Data analysis

All data were entered into the Statistical Package for the Social Sciences for Windows (Version 19.0, Chicago, IL, U.S.) for statistical analysis. The results of the questionnaire are expressed as frequency distributions and were computed in percentages. For discrete data, the chi square (χ2) test was used for comparison. For continuous data, an analysis of variance or Student’s t-test was used to compare the scores based on groups. A level of *P* < 0.05 was considered statistically significant for all analyses.

## Results

### Demographic information

A total of 1069 subjects fully completed the questionnaire. Fifty-six subjects did not complete the questionnaire. The sample included 1051 women (98.3%) and 18 men (1.7%). The average age of staff members was 40.1 ± 8.2 years. Teachers with health-care training accounted for 18.1% of the sample, and teachers without health-care training accounted for 81.9%. Of all participants, 82.3% had a bachelor’s degree or above, while 17.7% had attended only high school or below. Three percent of the staff had in medical-related degrees, and 57.3% majored in preschool education. Of the total sample, 29.5% had less than 10 years of teaching experience, 59.4% had more than 10 years but less than 30 years of teaching experience, and 11.1% had more than 30 years of teaching experience. The demographic characteristics of the staff are listed in Table 1.

**Table 1:** Demographic characteristics of the staff members (*N* = 1069)

Characteristics	^a^N (%)
Kindergarten category	
Public	847 (79.2%)
Other	222 (20.8%)
Staff member’s highest education level	
High school or below	189 (17.7%)
College or above	880 (82.3%)
Staff categories	
Had health-care training	193 (18.1%)
Did not have health-care training	876 (81.9%)
Teaching (years)	
< 10	315 (29.5%)
10–30	635 (59.4%)
> 30	119 (11.1%)
Major	
Medical related	33 (3.0%)
Preschool education	612 (57.3%)
Other	424 (39.7%)

^a^N denotes the number of valid answers

### Familiarity with epilepsy

Of the 1069 staff members who participated in the survey, 65.8% reported that they had heard about epilepsy, and 34.2% reported that they had never heard of epilepsy. Among the former group, only 36.2% knew about epilepsy because they had attended relevant training, 27.9% learned about it through public media and 13.9% had heard other people talking about it. In this survey, only 29.4% of the staff members had seen seizures, either in other adults or in preschool children, 18.9% of the staff had experience teaching children with epilepsy, and 17.6% of the staff had experience providing first aid during seizures, as seen in Table 2.

**Table 2:** Familiarity with epilepsy (*N =* 1069)

Item	^a^N (%)	^a^N (%)
	Yes	No
Had received epilepsy-related information or training	705 (65.8%)	366 (34.2%)
Source of the information		
Self-study	86 (8.1%)	983 (91.8%)
Public media	298 (27.9%)	771 (72.1%)
Doctors	71 (6.6%)	998 (93.4%)
Parents of children with epilepsy	78 (7.3%)	991 (92.7%)
In conversation	149 (13.9%)	920 (86.1%)
Attended relevant training	387 (36.2%)	682 (63.8%)
Had seen someone have a seizure previously	314 (29.4%)	755 (70.6%)
Experience with teaching a child with epilepsy	202 (18.9%)	867 (81.1%)
Experience with applying first-aid for seizures	188 (17.6%)	881 (82.4%)

^a^N denotes the number of valid answers

### Knowledge of epilepsy

None of the staff members surveyed answered all questions correctly. Regarding general knowledge about epilepsy, 17% of the staff considered epilepsy to be an infectious disease, and the difference between the answers of teachers with health-care training (12.8%) and teachers without health-care training (4.2%) was statistically significant (χ2 = 5.060, *p* = 0.029). A total of 69.3% of the staff members considered epilepsy to be a chronic brain disease and believed that it cannot be cured or controlled with medication (17% among teachers with health-care training, 52.3% among teachers without health-care training, χ2 = 2.204, *p* = 0.138).

The subjects lacked knowledge regarding first aid for seizures. When seeing children having seizures, only 17.6% of teachers knew how to provide appropriate first aid (31.6% of all teachers with health-care training, 14.5% of all teachers without health-care training, χ2 = 31.848, *p* < 0.001). Correct responses regarding first aid for seizures, such as laying the person on his or her side (24.9% in total; 44.0% of teachers with health-care training, 42.7% of teachers without health-care training, χ2 = 45.684, *p* < 0.001), moving harmful objects out of the way (20.7% in total; 30.6% of teachers with health-care training, 18.6% of teachers without health-care training, χ2 = 13.755, *p* < 0.001), protecting the head (36.1% in total; 48.2% of teachers with health-care training, 33.6% of teachers without health-care training, χ2 = 16.646, *p* < 0.001), waiting until the seizure ends (7.9% in total; 12.4% of teachers with health-care training, 6.8% of teachers without health-care training, χ2 = 6.816, *p* = 0.009), and dialing the emergency number (40.1% in total; 46.1% of teachers with health-care training, 39.4% of teachers without health-care training, χ2 = 2.971, *p* = 0.085) were low. There is no statistically significant difference in the level of epilepsy knowledge between urban and suburban staff. But there was a significant difference in the accuracy of first-aid knowledge between teachers with health-care training and teachers without health-care training. Most of the staff said they would apply first-aid techniques, but the techniques they selected were inappropriate, such as placing something in the person’s mouth (69% in total; 61.1% of teachers with health-care training, 70.8% of teachers without health-care training, χ2 = 6.870, *p =* 0.009) and attempting to adjust cramped limbs (9.8% in total; 9.8% of teachers with health-care training, 8.4% of teachers without health-care training, χ2 = 0.389, *p =* 0.533), seen in Table 3.

**Table 3:** General and first-aid knowledge about epilepsy (*N =* 1069)

Item	Teachers With healthcare training ^a^N (%) (*N* = 193)	Other ^a^N (%) (*N* = 876)	Total. ^a^N (%) (*N =* 1069)	Chi-square test value	*p*
**General knowledge about epilepsy**					
Epilepsy is an infectious disease	45 (23.3%)	137 (15.6%)	182 (17%)	5.060	0.029
Epilepsy is a chronic brain disease and cannot be cured or controlled	117 (57.9%)	452 (52.1%)	569 (69.3%)	2.204	0.138
**Accuracy of knowledge of first-aid for seizures**					
Have experience applying first-aid for seizures	61 (31.6%)	127 (14.5%)	188 (17.6%)	31.848	< 0.001
**Correct**					
Lay the child on his or her side	85 (44.0%)	182 (20.8%)	266 (24.9%)	45.684	< 0.001
Move harmful objects out of the way	59 (30.6%)	163 (18.6%)	221 (20.7%)	13.755	< 0.001
Protect the child’s head	93 (48.2%)	294 (33.6%)	386 (36.1%)	16.646	< 0.001
Wait until the seizure ends	24 (12.4%)	60 (6.8%)	84 (7.9%)	6.816	0.009
Administer related medicines	8 (4.1%)	34 (3.9%)	42 (3.9%)	0.029	0.864
Dial the 120 emergency number	89 (46.1%)	345 (39.4%)	433 (40.1%)	2.971	0.085
**Incorrect**					
Pull the child’s tongue	7 (3.6%)	26 (3.0%)	33 (3.1%)	0.230	0.632
Adjust cramped limbs	19 (9.8%)	74 (8.4%)	93 (8.7%)	0.389	0.533
Put something in the child’s mouth	118 (61.1%)	620 (70.8%)	738 (69%)	6.870	0.009

^a^N denotes the number of valid answers

### Attitudes towards epilepsy

The staff have different attitudes towards children with epilepsy. Most of the staff members had a positive attitude towards children with epilepsy: 70.1% believed that seizures are not dangerous for other children, 63.7% let their children play or survey with children with epilepsy, and 81.7% agreed that children with epilepsy can attend public school. However, some staff members had negative attitudes towards children with epilepsy: 70.8% of the staff members were afraid to have children with epilepsy in their classroom, 40.9% of the staff members thought that they should restrict the activities of children with epilepsy, and 48.8% of the staff members agreed that placing children with epilepsy in a dedicated classroom is advisable, as seen Table 4.

**Table 4:** Attitude toward epilepsy (*N* = 1069)

Item	N^a^ (%)	N^a^ (%)
**Attitude**	Yes	No
**Positive**		
Would let their child play or study with children with epilepsy	681 (63.7%)	388 (36.3%)
Most children with epilepsy can attend public school	873 (81.7%)	196 (18.3%)
Seizures are not dangerous for other children	749 (70.1%)	320 (29.9%)
**Negative**		
Would restrict the activities of children with epilepsy	437 (40.9%)	632 (59.1%)
Afraid having children with epilepsy in their classroom	757 (70.8%)	312 (29.2%)
Children with epilepsy should be placed in a dedicated classroom	522 (48.8%)	547 (51.2%)

^a^N denotes the number of valid answers

## Discussion

The purpose of this survey was to elaborate teachers’ knowledge and attitudes towards epilepsy and determine whether further epilepsy knowledge and first-aid training are required. Assessing teachers’ knowledge about epilepsy is an important issue in determining their attitudes towards children with epilepsy [20].

Overall staff knowledge about epilepsy is low. Epilepsy can be controlled with medication, but only 30.7% of the staff believe that epilepsy can be cured or controlled in this way. This rate was similar to the one found in survey in Togo [21], where only 30% of caregivers recognized that epilepsy could be cured by using medicines. However, Ibinga et al. reported that 42.8% of the staff in their survey thought that epilepsy could be treated by modern or traditional medicine [18]. In this survey, 17% of the staff in this survey thought that epilepsy is an infectious disease, however, a survey in Niger, epilepsy is considered to be a contagious disease by 46.2% of respondents [22]. This misconception among school teachers has an impact on teachers’ attitudes towards children with epilepsy, negative attitudes and beliefs about epilepsy of the school teachers can lead to the expulsion of the child and even the no schooling. Besides, some children with epilepsy may be rejected from their peers for fear of being contaminated [22]. The results of this survey indicate that the overall knowledge of staff members about epilepsy is lacking, as evidenced by the low but visible frequency of incorrect responses about how to work with children with epilepsy. When they have encountered a child experiencing a seizure in the past, only 17.6% of teachers applied first aid, which may be because the teachers lack knowledge about appropriate first-aid for seizures and fear legal consequences in the case of an accident. It is more common for teachers with health-care training to take appropriate first-aid measures than it is for teachers without health-care training to do so, which may be related to the former’s richer first-aid knowledge. In this survey, only 3.9% of teachers had administered the appropriate medicines to a child who was having a seizure. This is similar to the results of an Italian survey, which showed that only 9% of teachers administer such medication to children when they are having seizures [23]. In this survey, 40.1% of the staff members would choose to call an ambulance in the event of a child having a seizure, which is in keeping with other studies; another survey found both that 45% of the teachers would call an ambulance and that they did not have sufficient knowledge of the appropriate first aid [24]. In this survey, the rate of correct answers was particularly low (under 50%) for the questions about putting something inside the person’s mouth and epilepsy being a chronic brain disease that cannot be cured or controlled. These results are in line with those of studies conducted in Hong Kong [25], the USA [26], Korea [27], and France [28]. This survey determined that 69% of the staff would choose to put an object in the mouth of the child to prevent him or her from biting his or her tongue. The staff seemed to be unaware of the danger of putting an object between the teeth of a seizing child [18], and the rate was similar to that of a survey conducted in Germany [10]. In this survey, we compared the proportion of teachers with health-care training and teachers without health-care training who implemented the correct first-aid procedures during a seizure, and we found that it is more common for teachers with health-care training to implement the right measures, such as laying children on their side, than for other teachers to do so. This may be related to the teachers’ professional training regarding health-care. Dumeier et al. demonstrated that education may be useful in improving those issues, and the rate of teachers in their survey who would place something solid in a seizing child’s mouth decreased from 13 to 7% after a 40-min training session [29]. A significant difference was recorded in the school staff before and after the training in terms of availability to administer rescue drugs (52% before training versus 90% after the training) [30]. This shows that training on epilepsy knowledge is very useful. In some countries, including Italy [31], Nigeria [32], and Germany [29, 33], teachers have been trained on how to handle epileptic episodes, and the results have indicate that carrying out epilepsy knowledge training has a great effect on improving the appropriateness of teachers’ first-aid measures for seizures and their attitudes towards children with epilepsy.

In this survey, most of the staff members’ attitudes towards children with epilepsy were positive: 63.7% of the staff members allowed their children to survey and play with children with epilepsy, 70.1% of the staff members believed that seizures are not dangerous for other children, and 81.7% of the staff members agreed that most children with epilepsy can attend public school. These findings are similar to another survey that found that the attitudes of teachers towards the enrollment of children with epilepsy in regular schools were positive [18]. However, some staff members’ attitudes towards children with epilepsy were negative: 55.4% of teachers were afraid to have children with epilepsy in the classroom, and they wanted other students to come into the classroom only after the children with epilepsy were controlled or cured. Narita et al. reported that 43% of teachers reacted negatively to the possibility of “having epileptic children in their class” because the teachers fear that such children may have seizures in their classroom, and the teachers will not know what to do [24]. A total of 60.9% of staff members in this survey thought that it is necessary to restrict the activity of children with epilepsy. Another survey indicated that attitudes towards the participation of children with epilepsy in physical activities are particularly negative, as such children have often been advised against participating in sports and exercise out of fear, over protection, and ignorance [31]. Having a better attitude towards epilepsy may be related to exposure, as staff who had acquaintances who had experienced seizures were most likely to demonstrate a positive attitude towards individuals with epilepsy [24]. A survey in Moroccan showed that a better knowledge of the disease leads to positive attitudes toward children with epilepsy. There was also an improvement in the knowledge and attitude score among a large population of teachers with relatives/family members with epilepsy [34]. Teachers who had previously taught children with epilepsy tended to have more positive attitudes in this regard, letting them, for example, play with their own children. Staff members who had previously participated in related training also showed positive attitudes and, for example, let the children with epilepsy participate in activities. Teachers with health-care training are more likely to have a positive attitude and give appropriate first aid to children with seizures. In another survey of Koreans, Dumeier indicated that teachers who had appropriate knowledge about and familiarity with epilepsy showed more positive attitudes [35]. Teachers with health-care training are more receptive to having children with epilepsy in their classrooms. This survey showed that most teachers’ attitudes towards children with epilepsy were positive, but increasing their actual first-aid knowledge and skills regarding epilepsy remains necessary.

This survey results clearly demonstrate that there are grave deficiencies among preschool staff in Shanghai in terms of knowledge about and first aid skills related to children with epilepsy. The level of first-aid knowledge among such staff was low, and more effort should be made to increase epilepsy training for preschool staff members. The lack of formal and effective training in teacher preparation programs coupled with a lack of continuing education requirements is one possible explanation for these results [12]. Carrying out epilepsy knowledge training with preschool staff can improve their level of first aid knowledge regarding epilepsy and improve their attitudes towards children with epilepsy [29, 31–33]. Certainly, a good knowledge of epilepsy by school staff increases the children’s safety in the management of seizures [30]. There is an urgent need for effective measures to educate staff on epilepsy and give them training in relevant first-aid practices. During the period of Corona Virus Disease 2019 (COVID-19), it is suggested that the educational institutions should invite professional neuropediatricians to train the teachers and healthcare teachers on epilepsy knowledge online, so that the teachers in the kindergartens can master relevant epilepsy knowledge. Or it is suggested that kindergartens should invite neuropediatricians to record videos of epilepsy knowledge and distribute them to the teachers for watching and learning.

This survey has several limitations. First, the investigation of 1069 staff members in Shanghai is not representative of other parts of China because there are socioeconomic disparities between China’s western and eastern provinces. Second, we investigated the level of knowledge among staff members by a written, self-report questionnaire, and thus no practical skills could be tested in this setting and the answers were most likely subjective. Last, this local cross-sectional survey cannot be generalized to other countries.

## Conclusion

In this survey, some teachers had misunderstandings about epilepsy, and most of them lacked relevant first-aid knowledge. The level of epilepsy first-aid knowledge among preschool staff in Shanghai was low. Thus, it is necessary to carry out training with preschools staff on knowledge about epilepsy and relevant first-aid practices.

## Supplementary information

**Additional file 1:.** Knowledge and attitudes among preschools staff in Shanghai, China, regarding epilepsy of questionnaire

**Additional file 2:.** Knowledge and attitudes among preschools staff in Shanghai, China, regarding epilepsy of data

**Additional file 3:.** Knowledge and attitudes among preschools staff in Shanghai, China, regarding epilepsy of STROBE

**Additional file 4:.** Knowledge and attitudes among preschools staff in Shanghai, China, regarding epilepsy of editorial certificate

## Data Availability

The datasets used and analyzed during the current study are available from the corresponding author on reasonable request.
