# Supplementary material for: Knowledge and attitudes among preschools staff in Shanghai, China, regarding epilepsy
Source: BMC Pediatr. 2020 Oct 13;20:477. doi: 10.1186/s12887-020-02376-3 (PMC7550838; doi:10.1186/s12887-020-02376-3)
Supplement: Supplementary file 1 — Additional file 1:. Knowledge and attitudes among preschools staff in Shanghai, China, regarding epilepsy of questionnaire [file 12887_2020_2376_MOESM1_ESM.docx]

A number of questions on epilepsy knowledge and attitude will be asked, please choice the correct answer that you consider to be. Our survey is voluntary and anonymous, therefore you can set your mind at ease.

**G1. Have you ever heard or read about epilepsy?**

(1) Yes

(2) No

**G2. If so, where did you obtain knowledge?**

(1) Independent study

(2) Public media

(3) Doctors

(4) Parents of students with epilepsy

(5) In conversation

(6) Attended relevant training

**G3. Has anyone around you or kindergarten children experienced epilepsy?**

(1) Yes

(2) No

**G4. Have you ever had students with epilepsy in your classroom?**

(1) Yes

(2) No

**G5. Do you think epilepsy is a contagious disease?**

(1) Yes

(2) No

**G6. Do you think children with epilepsy have a higher mental incidence?**

(1) Yes

(2) No

**G7. Do you think that epilepsy is a chronic brain disease that cannot be cured or controlled?**

(1) Yes

(2) No

**G8.** **What do you think is the average intelligence of a person with epilepsy?**

(1) Normal intelligence

(2) Below average

(3) Above average

**G9. Are you afraid of having a student with epilepsy in your classroom and/or would prefer to have student’s epilepsy cured or under control before entering your classroom?**

(1) Yes

(2) No

**G10. Would you prefer to have all students with epilepsy placed in special classroom?**

(1) Yes

(2) No

**G11.** **Is it necessary to restrict the activities of children with epilepsy?**

(1) Yes

(2) No

**G12. Would you allow your child to play or sit in the same class with a child with epilepsy?**

(1) Yes

(2) No

**G13. Most children with epilepsy can go to public schools?**

(1) Yes

(2) No

**G14. Have you provided first aid to children with seizures?**

(1) Yes

(2) No

**G15. Which of the following methods did you take to provide first aid to children with epileptic seizures?**

(1) Lay the child on his or her side

(2) Dial the 120 emergency number

(3) Pull the child's tongue

(4) Prevent the child from spasm

(5) Administer related medicines

(6) Move harmful objects out of the way

(7) Protect the child’s head

(8) Wait until the seizure ends

(9) Stuff something into the child's mouth to prevent tongue bite

**G16. Children with epilepsy can be dangerous to other children during seizures?**

(1) Yes

(2) No
