# Supplementary material for: Knowledge and attitudes among preschools staff in Shanghai, China, regarding epilepsy
Source: BMC Pediatr. 2020 Oct 13;20:477. doi: 10.1186/s12887-020-02376-3 (PMC7550838; doi:10.1186/s12887-020-02376-3)
Supplement: Supplementary file 2 — Additional file 2:. Knowledge and attitudes among preschools staff in Shanghai, China, regarding epilepsy of data [file 12887_2020_2376_MOESM2_ESM.doc]

GET DATA /TYPE=XLS /FILE='C:\Users\qiuqiu\Desktop\ epilepsy questionnaire summary.xls' /SHEET=name '2019 reassessment' /CELLRANGE=full /READNAMES=on /ASSUMEDSTRWIDTH=32767. RECODE A10 (1=1) (2 thru 5=2) INTO health teacher. EXECUTE. RECODE A10 G5 (1=1) (2 thru 3=2) INTO health teacher infectious disease. EXECUTE. RECODE A10 G5 G7 (1=1) (2 thru 3=2) INTO health teacher infectious disease chronic encephalopathy. VARIABLE LABELS chronic encephalopathy'incurable'. EXECUTE. RECODE A10 G5 G7 G12 (1=1) (2 thru 3=2) INTO health teacher infectious disease chronic encephalopathy Play with children with epilepsy. VARIABLE LABELS chronic encephalopathy'incurable'. EXECUTE. CROSSTABS /TABLES=Health care teacher BY infectious diseases/FORMAT=AVALUE TABLES /STATISTICS=CHISQ /CELLS=COUNT ROW COLUMN TOTAL /COUNT ROUND CELL.
 
Data Set1

Health teachers interstitial tabs for infectious diseases.	
			infectious diseases		
			1.00	2.00	¦X计	
Health teachers	1.00	count	5	188	193	
		% of health teachers	2.6%	97.4%	100.0%	
		% in Infectious Diseases	6.8%	18.9%	18.1%	
		% of the total number	.5%	17.6%	18.1%	
	2.00	count	69	807	876	
		% of health teachers	7.9%	92.1%	100.0%	
		% in Infectious Diseases	93.2%	81.1%	81.9%	
		% of the total number	6.5%	75.5%	81.9%	
	Total	count	74	995	1069	
		% of health teachers	6.9%	93.1%	100.0%	
		% in Infectious Diseases	100.0%	100.0%	100.0%	
		% of the total number	6.9%	93.1%	100.0%	


Chi-square test	
	Numerical	df	Progressive Sig. (Two Sides)	Precision Sig.(two-sided)	Precision Sig.(one-sided)	
Pearson Chi-square	6.859a	1	.009			
Continuous correction b.	6.063	1	.014			
like an even ratio.	8.464	1	.004			
Fisher accurate testing				.007	.004	
						
	
a. 0 cell (.0%) The expected count is less than 5. The minimum expectation count is 13.36. b. Only for the 2x2 table.
	


CROSSTABS   /TABLES=health teachers BY Chronic encephalopathy /FORMAT=AVALUE TABLES   /STATISTICS=CHISQ   /CELLS=COUNT ROW COLUMN TOTAL   /COUNT ROUND CELL


Data Set2

Health teachers incurable cross-tabmaking	
			incurable		
			1.00	2.00	¦X计	
Health teachers	1.00	count	41	152	193	
		% of health teachers	21.2%	78.8%	100.0%	
		% in incurable	18.3%	18.0%	18.1%	
		% of the total number	3.8%	14.2%	18.1%	
	2.00	count	183	693	876	
		% of health teachers	20.9%	79.1%	100.0%	
		% in incurable	81.7%	82.0%	81.9%	
		% of the total number	17.1%	64.8%	81.9%	
	¦X计	count	224	845	1069	
		% of health teachers	21.0%	79.0%	100.0%	
		% in incurable	100.0%	100.0%	100.0%	
		% of the total number	21.0%	79.0%	100.0%	


Chi-square test	
	Numerical	df	Progressive Sig. (Two Sides)	Precision Sig.(two-sided)	Precision Sig.(one-sided)	
Pearson Chi-square	.012a	1	.913			
Continuous correction b.	.000	1	.991			
like an even ratio.	.012	1	.913			
Fisher accurate testing				.922	.491	
						
a. 0 cell (.0%) The expected count is less than 5. The minimum expectation count is 40.44.	
b. Only the 2x2 table is calculated.	


CROSSTABS   /TABLES=health teachers BY paly with CWE   /FORMAT=AVALUE TABLES   /STATISTICS=CHISQ   /CELLS=COUNT ROW COLUMN TOTAL   /COUNT ROUND CELL.


Data set3
Chi-square test	
	Numerical	df	Progressive Sig. (Two Sides)	Precision Sig.(two-sided)	Precision Sig.(one-sided)	
Pearson Chi-square	1.292a	1	.256			
Continuous correction b.	1.111	1	.292			
like an even ratio.	1.308	1	.253			
Fisher accurate testing				.282	.146	
						
a. 0 Cells (.0%) The expected count is less than 5. The minimum expectation count is 69.87.	
b. Only the 2x2 table is calculated. 	


CROSSTABS   /TABLES=health teachers BY G1501   /FORMAT=AVALUE TABLES   /STATISTICS=CHISQ   /CELLS=COUNT ROW COLUMN TOTAL   /COUNT ROUND CELL.


Data set4

Chi-square test	
	Numerical	df	Progressive Sig. (Two Sides)	Precision Sig.(two-sided)	Precision Sig.(one-sided)	
Pearson Chi-square	45.684a	1	.000			
Continuous correction b.	44.451	1	.000			
like an even ratio.	41.684	1	.000			
Fisher accurate testing				.000	.000	
						
a. Cell 0 (.0%) The expected count is less than 5. The minimum expectation count is 48.20.	
b. 2x2 tables are calculated.	


CROSSTABS   /TABLES=health teachers BY G1502   /FORMAT=AVALUE TABLES   /STATISTICS=CHISQ   /CELLS=COUNT ROW COLUMN TOTAL   /COUNT ROUND CELL.


Data set5


Chi-square test	
	Numerical	df	Progressive Sig. (Two Sides)	Precision Sig.(two-sided)	Precision Sig.(one-sided)	
Pearson Chi-square	2.971a	1	.085			
Continuous correction b.	2.698	1	.100			
like an even ratio.	2.943	1	.086			
Fisher accurate testing				.089	.051	
						
a. a. Cell 0 (.0%) The expected count is less than 5. The minimum expectation count is 78.36.	
b. 2x2 tables are calculated.	


CROSSTABS   /TABLES=health teachers BY G1505   /FORMAT=AVALUE TABLES   /STATISTICS=CHISQ   /CELLS=COUNT ROW COLUMN TOTAL   /COUNT ROUND CELL.


Data set6


Chi-square test	
	Numerical	df	Progressive Sig. (Two Sides)	Precision Sig.(two-sided)	Precision Sig.(one-sided)	
Pearson Chi-square	.029a	1	.864			
Continuous correction b.	.000	1	1.000			
like an even ratio.	.029	1	.865			
Fisher accurate testing				.838	.497	
						
a. Cell 0 (.0%) The expected count is less than 5. The minimum expectation count is 7.58.	
b. Only 2x2 tables are calculated.	


CROSSTABS   /TABLES=health teachers BY G1507   /FORMAT=AVALUE TABLES   /STATISTICS=CHISQ   /CELLS=COUNT ROW COLUMN TOTAL   /COUNT ROUND CELL.


Data set7


Chi-square test	
	Numerical	df	Progressive Sig. (Two Sides)	Precision Sig.(two-sided)	Precision Sig.(one-sided)	
Pearson Chi-square	14.646a	1	.000			
Continuous correction b.	14.020	1	.000			
like an even ratio.	14.249	1	.000			
Fisher accurate testing				.000	.000	
						
a. 0 Cells (.0%) The expected count is less than 5. The minimum expectation count is 69.87.	
b. Only 2x2 tables are calculated.	


CROSSTABS   /TABLES=health teachers BY G1508   /FORMAT=AVALUE TABLES   /STATISTICS=CHISQ   /CELLS=COUNT ROW COLUMN TOTAL   /COUNT ROUND CELL.


Data set8


Chi-square test	
	Numerical	df	Progressive Sig. (Two Sides)	Precision Sig.(two-sided)	Precision Sig.(one-sided)	
Pearson Chi-square	6.816a	1	.009			
Continuous correction b.	6.066	1	.014			
like an even ratio.	6.093	1	.014			
Fisher accurate testing				.012	.009	
						
a. 0 Cells (.0%) The expected count is less than 5. The minimum expectation count is 15.17.	
b. Only 2x2 tables are calculated.	


CROSSTABS   /TABLES=health teachers BY G1509   /FORMAT=AVALUE TABLES   /STATISTICS=CHISQ   /CELLS=COUNT ROW COLUMN TOTAL   /COUNT ROUND CELL.

Data set9

Chi-square test	
	Numerical	df	Progressive Sig. (Two Sides)	Precision Sig.(two-sided)	Precision Sig.(one-sided)	
Pearson Chi-square	6.870a	1	.009			
Continuous correction b.	6.427	1	.011			
like an even ratio.	6.660	1	.010			
Fisher accurate testing				.010	.006	
						
a. 0 Cells (.0%) The expected count is less than 5. The minimum expectation count is 59.76.	
b. Only 2x2 tables are calculated.	


CROSSTABS   /TABLES=health teachers BY G1503   /FORMAT=AVALUE TABLES   /STATISTICS=CHISQ   /CELLS=COUNT ROW COLUMN TOTAL   /COUNT ROUND CELL.


Data set10

Chi-square test	
	Numerical	df	Progressive Sig. (Two Sides)	Precision Sig.(two-sided)	Precision Sig.(one-sided)	
Pearson Chi-square	.230a	1	.632			
Continuous correction b.	.062	1	.803			
like an even ratio.	.220	1	.639			
Fisher accurate testing				.645	.385	
						
a. Cell 0 (.0%) The expected count is less than 5. The minimum expectation count is 5.96.	
b. Only 2x2 tables are calculated.	


CROSSTABS   /TABLES=health teachers BY G1504   /FORMAT=AVALUE TABLES   /STATISTICS=CHISQ   /CELLS=COUNT ROW COLUMN TOTAL   /COUNT ROUND CELL.

Data set11

Chi-square test	
	Numerical	df	Progressive Sig. (Two Sides)	Precision Sig.(two-sided)	Precision Sig.(one-sided)	
Pearson Chi-square	.389a	1	.533			
Continuous correction b.	.233	1	.630			
like an even ratio.	.377	1	.539			
Fisher accurate testing				.572	.308	
						
a. 0 Cells (.0%) The expected count is less than 5. The minimum expectation count is 16.79.	
b. Only 2x2 tables are calculated.	


CROSSTABS   /TABLES=health teachers BY G1506   /FORMAT=AVALUE TABLES   /STATISTICS=CHISQ   /CELLS=COUNT ROW COLUMN TOTAL   /COUNT ROUND CELL.


Data set12

Chi-square test	
	Numerical	df	Progressive Sig. (Two Sides)	Precision Sig.(two-sided)	Precision Sig.(one-sided)	
Pearson Chi-square	13.755a	1	.000			
Continuous correction b.	13.037	1	.000			
like an even ratio.	12.780	1	.000			
Fisher accurate testing				.000	.000	
						
a. 0 Cells (.0%) The expected count is less than 5. The minimum expectation count is 40.08.	
b. Only 2x2 tables are calculated.	
