# Supplementary material for: Knowledge and attitudes among preschools staff in Shanghai, China, regarding epilepsy
Source: BMC Pediatr. 2020 Oct 13;20:477. doi: 10.1186/s12887-020-02376-3 (PMC7550838; doi:10.1186/s12887-020-02376-3)
Supplement: Supplementary file 4 — Additional file 4:. Knowledge and attitudes among preschools staff in Shanghai, China, regarding epilepsy of editorial certificate [file 12887_2020_2376_MOESM4_ESM.docx]

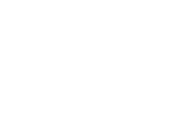
Editing Certificate

This document certifies that the manuscript


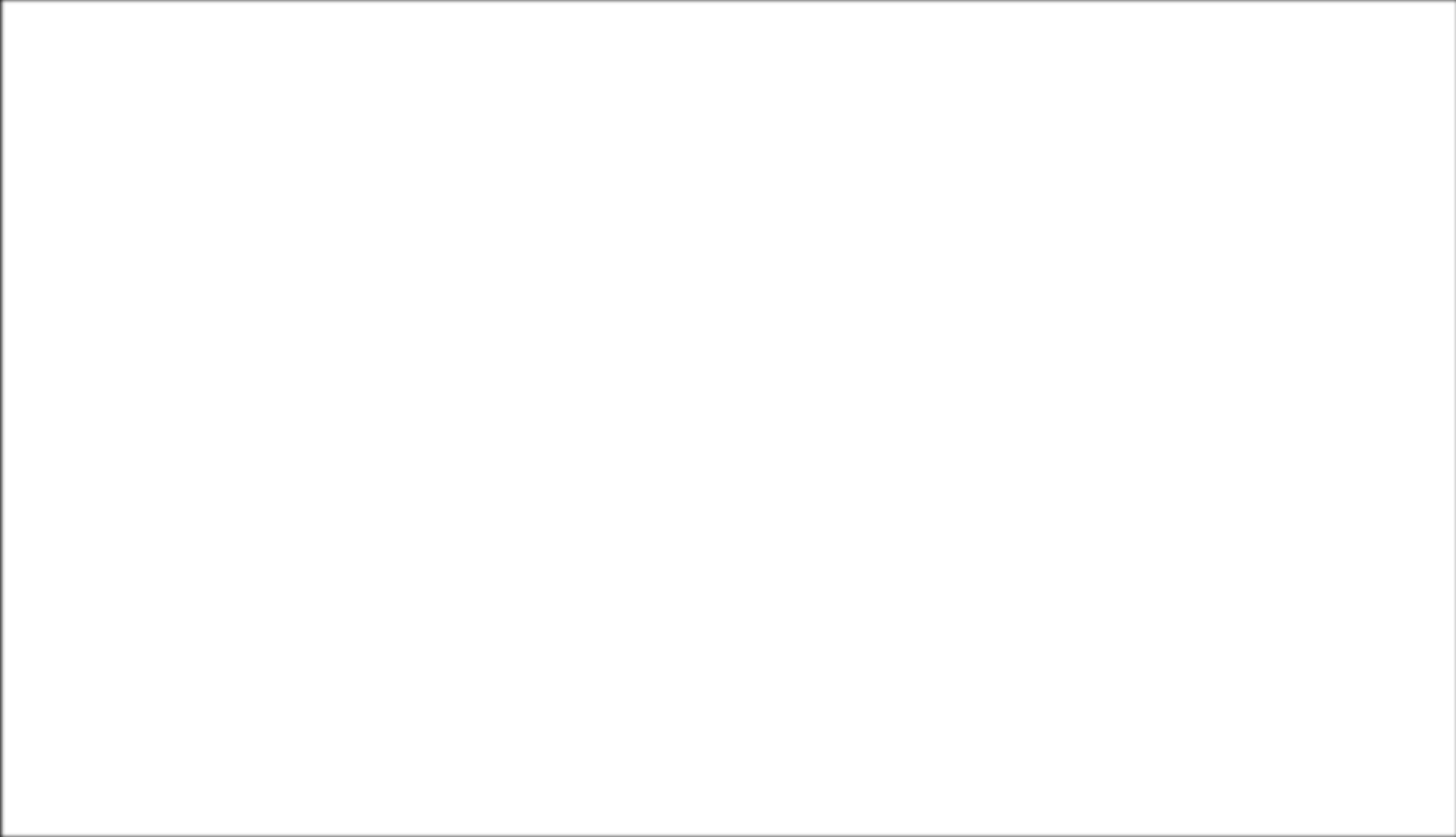

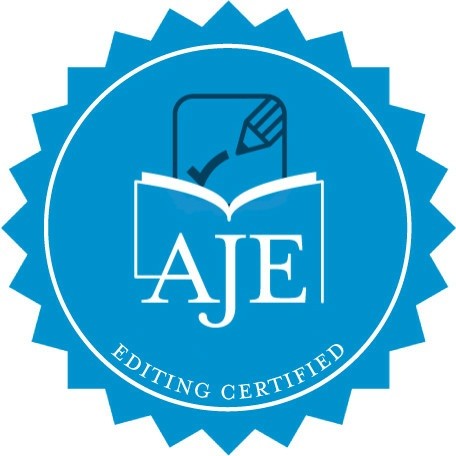


# Knowledge and attitudes of epilepsy among preschool staff in Shanghai, China, regarding epilepsy

prepared by the authors

# Liyan Qiu, Lixiao Shen, Junli Wang, Fang Ren, Mingyu Xu, Fan Jiang, Xiaoyang Sheng, Fei Li, Feng Li

was edited for proper English language, grammar, punctuation, spelling, and overall style by one or more of the highly qualified native English speaking editors at AJE.

This certificate was issued on **June 2, 2020** and may be verified

on the [AJE website](https://www.aje.com/certificate) using the verification code **CB70-793F-ECE1-154D-B78P** .

Neither the research content nor the authors' intentions were altered in any way during the editing process. Documents receiving this certification should be English-ready for publication; however, the author has the ability to accept or reject our suggestions and changes. To verify the final AJE edited version, please visit our verification page at [aje.com/certificate](https://www.aje.com/certificate).

If you have any questions or concerns about this edited document, please contact AJE at [support@aje.com.](mailto:support@aje.com)

AJE provides a range of editing, translation, and manuscript services for researchers and publishers around the world.

For more information about our company, services, and partner discounts, please visit [aje.com](https://www.aje.com/).
